# Analysis of chromosomal abnormalities by CGH-array in patients with dysmorphic and intellectual disability with normal karyotype

**DOI:** 10.1590/S1679-45082016AO3592

**Published:** 2016

**Authors:** Rodrigo Pratte-Santos, Katyanne Heringer Ribeiro, Thainá Altoe Santos, Terezinha Sarquis Cintra

**Affiliations:** 1 Universidade Federal do Espírito Santo, Vitória, ES, Brazil.; 2 Faculdade de Ciências Biomédicas do Espírito Santo, Cariacica, ES, Brazil.; 3 Laboratório de Genética do Espírito Santo, Vitória, ES, Brazil.

**Keywords:** Chromosome aberrations, Comparative genomic hybridization/methods, Karyotype, Body dysmorphic disorders, Intellectual disability

## Abstract

**Objective:**

To investigate chromosomal abnormalities by CGH-array in patients with dysmorphic features and intellectual disability with normal conventional karyotype.

**Methods:**

Retrospective study, carried out from January 2012 to February 2014, analyzing the CGH-array results of 39 patients.

**Results:**

Twenty-six (66.7%) patients had normal results and 13 (33.3%) showed abnormal results - in that, 6 (15.4%) had pathogenic variants, 6 (15.4%) variants designated as uncertain and 1 (2.5%) non-pathogenic variants.

**Conclusion:**

The characterization of the genetic profile by CGH-array in patients with intellectual disability and dysmorphic features enabled making etiologic diagnosis, followed by genetic counseling for families and specific treatment.

## INTRODUCTION

Chromosome anomalies are associated with a spectrum of clinical characteristics, which include primarily facial dysmorphism, intellectual disability (ID), microcephaly, intrauterine growth retardation, neuropsychiatric alterations, and congenital cardiopathies.^1^

Intellectual disability is characterized by lower than average intelligence or mental capacity, and by the lack of abilities needed for day-to-day living. People with ID may learn new skills, but they do so more slowly. There are several grades of ID, ranging from mild to severe.^2^ In the general population, 2 to 3% of individuals have some form of ID, and chromosomal anomalies are detected in 4 to 28% of these cases, depending on the selection of patients and sensitivity of the techniques used. However, the use of more recent methodologies has shown that 10 to 25% of ID cases involved very small subtelomeric or interstitial rearrangements. The clinical consequences of a chromosome rearrangement are generally related to its location, size, and the quantity of genes involved and their function.^3^ Patients with suspected chromosome anomalies are initially indicated for the karyotype test, with G-banding, a conventional cytogenetic technique. Chromosomes are analyzed microscopically in metaphase with a resolution of 400 to 550 bands. However, this level of resolution does not detect chromosome alterations that affect segments smaller than 5Mb. In average, 15 to 20% of individuals with ID, autism spectrum disorders, and multiple congenital anomalies are diagnosed with the methodology of array-base comparative genomic hybridization (aCGH).^4^

The aCGH technique offers important advantages over the methods of conventional cytogenetics, since besides not requiring a cell culture, analysis of the DNA extracted from different types of paraffin-embedded tissues is possible. Moreover, it is possible to investigate, in a single analysis, thousands of chromosome regions, detecting submicroscopic chromosome losses and gains in a single test. The aCGH test allows the clinical diagnosis of chromosome abnormalities at a high resolution and represents the conventional genetic and molecular integration.^5^ The aCGH method can be applied to detect changes in the number of variant copies (CNV, *copy-number variations*) at a resolution as low as 1Kb of matrixes in the genome. This method has been increasingly indicated for evaluation of individuals with dysmorphic characteristics and ID. The aCGH method is considered a first-line test and suggests the chromosome analysis of G bands for specific cases, such as patients with obvious chromosome syndromes, such as Down syndrome and familial history of chromosome rearrangements.^6^

## OBJECTIVE

To evaluate chromosome abnormalities by aCGH in individuals with dysmorphisms and intellectual disability with a normal karyotype.

## METHODS

This is a retrospective study conducted at the *Laboratório de Genética do EspíritoSanto*, located in Vitória, capital of the State of Espírito Santo, Brazil. This unit cared for an average of 15 patients a day, and 75% had genetic diseases. The aCGH data of 39 patients were selected who had normal results of conventional karyotype analysis, during the period of January 2012 and February 2014, with suspected genomic microalterations.

The age range was 3-22 years, and the ratio males and females was 8:5, respectively. The inclusion criteria were individuals who presented with ID and/or dysmorphisms, had normal results of their karyotype or G band tests, and underwent the aCGH test. The individuals with altered karyotypes were excluded from the study.

The aCGH analysis was performed using the SurePrint G3 Human CGH Microarray Kit, 4X180K (Agilent Technologies, CA, United States), following the manufacturer’s instructions. The analysis was carried out by the method of preparation of samples, using oligonucleotides coupled in synthetic DNA microarrays. Digestion was done with a restriction enzyme, followed by a sequence of adaptors for each fragment, allowing amplification of multiple *loci* and using a single supplementary initiator for these adapters. Agilent CytoGenomics (Agilent Technologies, CA, United States) software was used to visualize, detect, and analyze chromosome patterns within the microarray profiles*.* ^7^ The classification of CNV detected in the aCGH test was obtained from information deposited in public international databases, such as GeneCards^**®**^ (http://www.genecards.org), MedGen (http://www.ncbi.nlm.nih.gov/medgen), ClinVar (http://www.ncbi.nlm.nih.gov/clinvar), Database of Genomic Variants (http://dgv.tcag.ca), and Database of Chromosomal Imbalance and Phenotype in Humans Using Ensemble Resources (DECIPHER; http://decipher.sanger.ac.uk).

This research was approved by the Research Ethics Committee of the *Hospital Meridional*, under CAAE: 43513215.9.0000.5070, Official Opinion number 1.008.131/2015. The patients evaluated were instructed as to the objective of the investigation and, upon agreement to participate in the study, signed the Informed Consent Form.

## RESULTS

**Figure 1:**
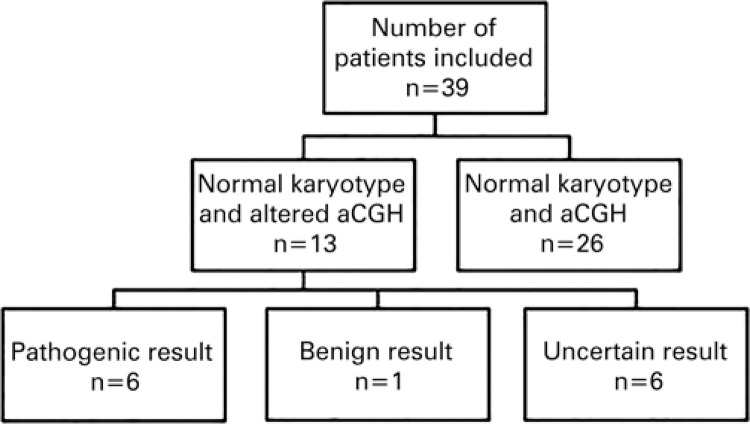
Flowchart of the results obtained by conventional karyotype and aCGH techniques

DNA samples from peripheral blood of 39 individuals with delayed intellectual development and dysmorphism were analyzed. Of these, 26 (66.7%) samples were excluded for not meeting the inclusion criteria. In all these 26 individuals, both the conventional karyotype test and the aCGH showed results considered normal. Of the total number of patients investigated, 13 (33.3%) were included in the study, that is, they presented with normal karyotypes and the aCGH had some form of alteration. With the results obtained, chromosome abnormalities were identified and the CNV were classified into three categories: pathogenic, benign, and of uncertain significance. Six (15.4%) patients presented with alterations classified as pathogenic; six (15.4%), with alterations of uncertain clinical significance; and one (2.6%) patient, was benign (Figure 1).

**Figure 2:**
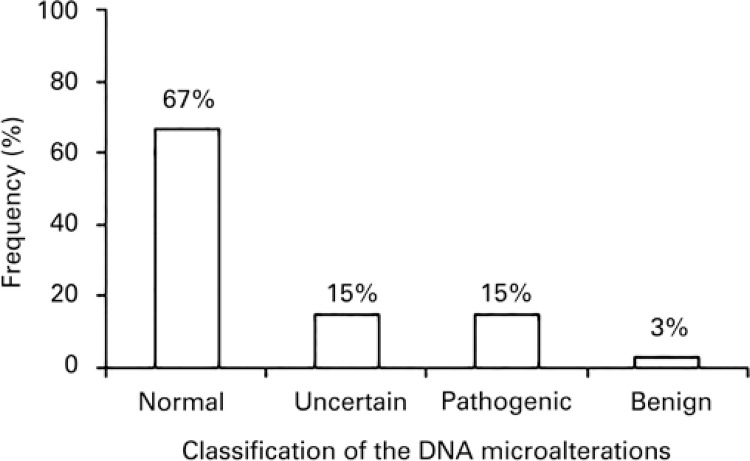
Results of the aCGH test in patients with intellectual disability and dysmorphisms and normal karyotypes

Pathogenic alterations were noted in 15.4% of cases (Figure 2). These results still have not been described in cytogenetic databases.

Chart 1 describes in detail the patients studied and their respective CNV. The individuals described with numbers 1, 4, 6, 7, 12, and 13, had variants with uncertain clinical significance; individuals with numbers 2, 3, 5, 8, 10, and 11 presented with pathogenic variants; and one single patient (9) presented with a variant classified as benign.

**Chart 1:** Data of patients with DNA microalterations by aCGH technique

Patient	Age (years)	Gender	Microalteration	Position of the alteration
1	9	F	arr [GRCh37]13q13.3	(38.838.148-39.175.432)x1
2	22	F	arr 10q26.3	(131.188.376-135.253.581)x1
3	6	F	arr 1q21.1	(145.388.369-145.955.098)x3
4	18	M	arr 4p15.32	(16.833.226-17.799.723)x3
5	3	M	arr[hg18] 1q43-q44	(239.332.947-247.179.289)x1
6	9	M	arr 22q12.3	(33.993.901-34.043.733)x1
7	14	M	arr 12p11.23	(27.197.557-27.651.334)x1
8	3	M	arr 6q25.2-q25.3	(155.486.120-158.262.536)x1
9	9	F	arr[GRCh37] Xp22.33	(398,974-697,436)x3
10	8	M	arr 2p24.3-p24.1	(13.844.661-23.659.168)x1
11	4	M	arr 8p23.1	(8.111.100-11.907.856)x1
12	5	F	arr 11p11.2	(48.131.676-48.783.109)x1
13	6	M	arr[GRCh37]13q12.11	(20.181.114-20.600.888)x3

F: female; M: male.

## DISCUSSION

Literature reports that the power of diagnosis of the aCGH test is roughly 10 to 20% in some disorders, such as autism, ID, congenital malformations, and several types of neoplasms. However, with conventional techniques, only 3 to 5% of these abnormalities would be detected.^8^ In the present study, the frequency of detection of microalterations by aCGH coincided with that of literature, that is, 15.4%.

The CNV are originated primarily by errors in DNA replication and by spontaneous and/or induced mutations. These alterations are common in individuals, and therefore there is no direct relation between their presence in the genome and symptoms.^9^ Thus, the quantity of these variants in DNA does not determine the degree of impairment caused by the disease.

In this study, each one of the six (15.4%) individuals presented with only a single CNV alteration classified as pathogenic, which was sufficient for determining the cause of the disease. The CNV could be classified as pathogenic or not, depending on the type of microduplication or microdeletion, containing genes in their region and depending on their size.^10^ In analyzing the aCGH data, one should verify if the CNVs are susceptible to being benign, pathogenic, or with unknown or uncertain clinical significance. The CNV that overlap critical regions of known syndromes are susceptible to being pathogenic in nature.^11^

Generally, duplications are less severe alterations than genome deletions, so that the absence of CNV is probably more pathogenic. The elimination of the CNV can be more prevalent in individuals with genomic disorders, and thus, confers a greater potential of risk for pathogenicity of the syndrome when observed as a *de novo* alteration in an affected individual.^12^

In the present study, the aCGH test was carried out in patient 8, and a chromosome microalteration was detected in the long arm of chromosome 6, the deletion of an interstitial segment of 2.8Mb, in 6q25.2-q25.3. The segment affected in chromosome 6 contains several genes, and deletions that overlap this same genome segment have already been described in other patients with variable clinical picture. The detected deletion included, among others, the gene ARID1B (614556), associated with ID.^13^

When observing a CNV with uncertain clinical significance, one should first evaluate if it is inherited or *de novo*. The parents’ test is very important to determine pathogenicity of most of CNV, and materials of the patient and parents should be compared. This gives an indication as to whether the CNV is inherited or not. Whenever possible, studies with cytogenetic tests should be considered, since they can provide information about the chromosome distribution and the risk of recurrence.^14^

The CNV with uncertain significance are not observed in the normal population and have poorly known functional genes or non-coding RNA, and their interpretation is still a challenge to researchers.^15^ Within this context, the result of the aCGH test of patient 1 showed the presence of a CNV with loss of a segment of 337 Kb of the long arm of chromosome 13, at 13q13.3. There are no descriptions of similar alterations at this genome segment in individuals in the general population. Hence, it is not possible to affirm that the variant detected is responsible for the clinical picture, which belongs to a category of variants designated in literature as having yet uncertain significance. The result of the test in patient 1 excluded as cause of the clinical picture the several syndromes of genome microdeletions or microduplications described in literature.

Analyzing the results of patients 6 and 7, smaller alterations were found than those possible of being detected by optic microscopy, in a conventional karyotype test. In the first case, a deletion of the interstitial segment of 49Kb of the long arm of chromosome 22 was observed at 22q12.3. In the second case, we noted a chromosome variant of 450Kb, a result of an interstitial deletion of the short arm of chromosome 12, at 12p11.23. In the literature, both microalterations are considered as having uncertain clinical significance.

Furthermore, in the classification of CNV, those determined as benign do not have clinical significance for the patient’s phenotype and appear in approximately 6% of human genome. There are genes in the genome with an ample variety relative to their size and the number of repetitions, so that patients with a normal clinical picture can present with a greater number of copies, making the interpretation of results very difficult.^16^ One of the patients presented with an interstitial deletion of 644Kb at 10q21.1. This deletion did not affect known genes, and was described as a benign alteration, causing no deleterious effects to the individual.

The growing quantity of information collected by the different databases should allow establishing a relation between a given CNV and a possible pathogenic condition with increased precision.^15^

In the present study, a greater number of microdeletions was found compared to that of microduplications. It is more likely that deletions in the genome are caused by an altered phenotype than that of the duplications. Microduplications usually cause a milder phenotype in patients.^17^

In cases of patients with delayed psychomotor development or mental disability, in whom the karyotype test was normal, we recommend the use of the aCGH technique to help making diagnosis. Considering this methodology is complex, the interpretation of results may be difficult, in some cases. Therefore, we recommend interpreting results with the support of a professional specialized in genetics, by means of genetic counseling.^18^

## CONCLUSION

The analysis by aCGH enabled detecting a considerable number of chromosome anomalies that had not been identified by conventional analysis using chromosome banding, or the karyotype test.

In this study, characterization of the genetic profile by aCGH in patients with intellectual disability and dysmorphism led to the etiologic diagnosis.

Despite the difficult interpretation, aCGH proved a sensitive technique that can be used as supplementary method in diagnosis of genetic diseases. This diagnosis is important in patients with rare or unknown etiologies.
