# Supplementary material for: Analysis of chromosomal abnormalities by CGH-array in patients with dysmorphic and intellectual disability with normal karyotype
Source: Einstein (Sao Paulo). 2016 Jan-Mar;14(1):30–4. doi: 10.1590/S1679-45082016AO3592 (PMC4872914; doi:10.1590/S1679-45082016AO3592)
Supplement: Translation

# Translation: Análise de anomalias cromossômicas por CGH-*array* em pacientes com dismorfias e deficiência intelectual com cariótipo normal

**Authors:** Rodrigo Pratte-Santos, Katyanne Heringer Ribeiro, Thainá Altoe Santos, Terezinha Sarquis Cintra

**Affiliations:** 1 Universidade Federal do Espírito Santo, Vitória, ES, Brasil.; 2 Faculdade de Ciências Biomédicas do Espírito Santo, Cariacica, ES, Brasil.; 3 Laboratório de Genética do Espírito Santo, Vitória, ES, Brasil.

**Keywords:** Aberrações cromossômicas, Hibridização genômica comparativa/métodos, Cariótipo, Transtornos dismórficos corporais, Deficiência intelectual

## Abstract

**Objetivo:**

Avaliar microalterações cromossômicas por CGH-*array* em portadores de dismorfias e deficiência intelectual com cariótipo normal.

**Métodos:**

Estudo retrospectivo, realizado no período de janeiro de 2012 a fevereiro de 2014, analisando os resultados de CGH-*array* de 39 pacientes.

**Resultados:**

Apresentaram resultados normais 26 (66,7%) pacientes; 13 (33,3%) tiveram resultados alterados, a saber: 6 (15,4%) com variantes patogênicas, 6 (15,4%) com variantes pertencentes à categoria designada como incerta, e 1 (2,5%) com variantes não patogênicas.

**Conclusão:**

A caracterização do perfil genético por CGH-*array* nos pacientes com deficiência intelectual e dismorfias possibilitou complementar o diagnóstico etiológico, permitindo a realização do aconselhamento genético para as famílias e tratamento específico.

## INTRODUÇÃO

As anomalias cromossômicas estão associadas a um espectro de características clínicas, que incluem principalmente dismorfia facial, deficiência intelectual (DI), microcefalia, retardo do crescimento intrauterino, alterações neuropsiquiátricas e cardiopatias congênitas.^1^

A DI caracteriza-se pela inteligência ou capacidade mental abaixo da média, e pela falta de habilidades necessárias para a vida do dia a dia. As pessoas com DI podem aprender novas habilidades, mas de forma mais lenta. Existem vários graus de DI, de leve a profundo.^2^ Na população em geral, 2 a 3% das pessoas são portadoras de DI, sendo que anomalias cromossômicas são detectadas em 4 a 28% desses casos, dependendo da seleção dos pacientes e da sensibilidade das técnicas empregadas. No entanto, o uso de metodologias mais recentes evidenciou que 10 a 25% dos casos de DI envolvem rearranjos muito pequenos, subteloméricos ou intersticiais. As consequências clínicas de um rearranjo cromossômico estão geralmente relacionadas com sua localização, seu tamanho, e a quantidade de genes envolvidos e sua função.^3^ Pacientes com suspeita de anomalias cromossômicas inicialmente são indicados para a realização do exame cariótipo com bandeamento G, técnica de citogenética convencional. Os cromossomos são analisados microscopicamente em metáfase com resolução de 400 a 550 bandas. Porém, esse nível de resolução não detecta alterações cromossômicas que afetam segmentos menores que 5Mb. Em média 15 a 20% dos indivíduos com DI, distúrbios do espectro do autismo e anomalias congênitas múltiplas são diagnosticados pela metodologia de hibridização genômica comparativa em *array* (aCGH).^4^

A técnica de aCGH oferece importantes vantagens sobre os métodos de citogenética convencional, pois, além de não ser necessária a obtenção de cultura celular, é possível a análise do DNA extraído de diferentes tipos de tecidos em parafina e há a capacidade de investigar, em uma única análise, milhares de regiões cromossômicas, detectando perdas e ganhos cromossômicos submicroscópicos em um único exame. O exame aCGH permite o diagnóstico clínico das anormalidades cromossômicas em uma alta resolução e representa a integração da genética convencional e a molecular.^5^ Pode-se aplicar o método de aCGH para a detecção de alterações no número de cópias variantes (CNV - *copy-number variations*) em uma resolução tão baixa quanto 1Kb de matrizes no genoma. Esse método vem sendo cada vez mais indicado para a avaliação de indivíduos com características dismórficas e DI. O uso de aCGH tem o peso de um teste de primeira linha e sugere a análise cromossômica de bandas G para casos específicos, como pacientes com síndromes cromossômicas óbvias, tais como síndrome de Down, e história familiar de rearranjos cromossômicos.^6^

## OBJETIVO

Avaliar microalterações cromossômicas por aCGH em portadores de dismorfias e deficiência intelectual com cariótipo normal.

## MÉTODOS

Estudo retrospectivo conduzido no Laboratório de Genética do Espírito Santo, situado em Vitória, capital do Estado do Espírito Santo, Brasil. Essa unidade atendia em média 15 pacientes diariamente, e 75% eram portadores de doenças genéticas. Foram selecionados os resultados de aCGH de 39 pacientes que possuíam os resultados da análise de cariótipo convencional normal, no período de janeiro de 2012 e fevereiro de 2014 com suspeitas de microalterações no genoma.

A idade dos pacientes incluídos no estudo variou entre 3 a 22 anos, e a proporção dos gêneros masculino e feminino foi de 8:5, respectivamente. Os critérios de inclusão foram indivíduos que apresentavam DI e/ou dismorfias, que tiveram o resultado do exame de cariótipo com bandeamento G normal, e que realizaram o teste de aCGH. Os indivíduos que apresentaram cariótipo alterado foram excluídos do estudo.

A análise de aCGH foi realizada utilizando o SurePrint G3 Human CGH Microarray Kit, 4X180K (Agilent Technologies CA, Estados Unidos), seguindo as instruções do fabricante*.* A análise foi realizada pelo método de preparação de amostras, utilizando oligonucleotídeos acoplados em *microarrays* de DNA sintético. Foi realizada digestão com enzima de restrição, seguida de uma sequência de adaptadores para cada fragmento, permitindo amplificar múltiplos *loci* e utilizando um único iniciador complementar a esses adaptadores. O *software Agilent CytoGenomics* (Agilent Technologies, CA, Estados Unidos) foi utilizado para visualizar, detectar e analisar padrões cromossômicos dentro dos perfis de *microarrays.* ^7^ A classificação das CNV detectadas no exame de aCGH foi obtidas a partir de informações depositadas em bancos de dados internacionais públicos, como GeneCards^**®**^ (http://www.genecards.org), MedGen (http://www.ncbi.nlm.nih.gov/medgen), ClinVar (http://www.ncbi.nlm.nih.gov/clinvar), *Database of Genomic Variants* (http://dgv.tcag.ca) e *Database of Chromosomal Imbalance and Phenotype in Humans Using EnsembleResources* (DECIPHER; http://decipher. sanger.ac.uk).

Esta pesquisa foi aprovada pelo Comitê de Ética em Pesquisa do Hospital Meridional, sob o número do CAAE: 43513215.9.0000.5070, parecer 1.008.131/2015. Os pacientes avaliados foram instruídos sobre os objetivos da pesquisa e, ao aceitarem participar do estudo, assinaram um Termo de Consentimento Livre e Esclarecido.

## RESULTADOS

Foram analisadas amostras de DNA de sangue periférico de 39 indivíduos com atraso no desenvolvimento intelectual e dismorfias. Destas, 26 (66,7%) amostras foram excluídas, pois não estavam de acordo com os critérios de inclusão da pesquisa. Em todos esses 26 indivíduos, tanto o exame de cariótipo convencional como o aCGH obtiveram resultados considerados normais. Do total de pacientes investigados, 13 (33,3%) foram incluídos no estudo, ou seja, apresentaram cariótipo normal e aCGH com algum tipo de alteração. Com os resultados obtidos, foram identificadas alterações cromossômicas e as CNV foram classificadas em três categorias: patogênicas, benignas e de significância incerta. Seis (15,4%) pacientes apresentaram alterações classificadas como patogênicas; seis (15,4%), alterações com significado clínico incerto; e um (2,6%) paciente, benigna (Figura 1).

**Figura 1:** Fluxograma dos resultados obtidos pelas técnicas de cariótipo convencional e aCGH

Foram observadas alterações patogênicas em 15,4% dos casos (Figura 2). Esses resultados ainda não foram descritos em bancos de dados citogenéticos.

**Figura 2:** Resultados de exame de aCGH em pacientes com deficiência intelectual e dismorfias que possuíam cariótipo normal

O quadro 1 descreve detalhadamente os pacientes estudados e suas respectivas variantes CNV apresentadas. Os indivíduos descritos com a numeração 1, 4, 6, 7, 12 e 13 possuíam variantes com significado clínico incerto; os indivíduos 2, 3, 5, 8, 10 e 11 apresentaram variante patogênicas; e uma única paciente (9) investigada apresentou variante classificada como benigna.

**Quadro 1:** Dados dos pacientes com resultado de microalteração no DNA por meio da técnica de aCGH

Paciente	Idade (anos)	Gênero	Microalteração	Posição da alteração
1	9	F	arr [GRCh37]13q13.3	(38.838.148-39.175.432)x1
2	22	F	arr 10q26.3	(131.188.376-135.253.581)x1
3	6	F	arr 1q21.1	(145.388.369-145.955.098)x3
4	18	M	arr 4p15.32	(16.833.226-17.799.723)x3
5	3	M	arr[hg18] 1q43-q44	(239.332.947-247.179.289)x1
6	9	M	arr 22q12.3	(33.993.901-34.043.733)x1
7	14	M	arr 12p11.23	(27.197.557-27.651.334)x1
8	3	M	arr 6q25.2-q25.3	(155.486.120-158.262.536)x1
9	9	F	arr[GRCh37] Xp22.33	(398,974-697,436)x3
10	8	M	arr 2p24.3-p24.1	(13.844.661-23.659.168)x1
11	4	M	arr 8p23.1	(8.111.100-11.907.856)x1
12	5	F	arr 11p11.2	(48.131.676-48.783.109)x1
13	6	M	arr[GRCh37]13q12.11	(20.181.114-20.600.888)x3

F: feminino; M: masculino.

## DISCUSSÃO

A literatura relata que o poder de diagnóstico do aCGH gira em torno de 10 a 20% em enfermidades como autismo, DI, malformações congênitas e vários tipos de neoplasias, sendo que, em técnicas convencionais, somente 3 a 5% dessas anormalidades seriam detectadas.^8^ No presente estudo, a frequência de detecções de microalterações por aCGH coincidiu com a literatura, ou seja, 15,4%.

As CNV são originadas principalmente por erros na replicação do DNA e por mutações espontâneas e/ou induzidas. Essas alterações são comuns nos indivíduos, não apresentando, portanto, relação direta de sua presença no genoma com os sintomas.^9^ Assim, a quantidade dessas variantes no DNA não determina o grau de comprometimento da doença.

No presente estudo, seis (15,4%) indivíduos apresentaram, cada um, somente uma única alteração CNV classificada como patogênica, que foram suficientes para determinar a causa da doença. As CNV podem ser classificadas como patogênicas ou não, do tipo microduplicação ou microdeleção, contendo genes em sua região e mediante seu tamanho.^10^ Ao analisar os dados de aCGH, deve-se verificar se as CNV são suscetíveis de serem benignas, patogênicas ou de significado clínico desconhecido ou incerto. As CNV que se sobrepõem a regiões críticas de síndromes conhecidas são suscetíveis de ser de natureza patogênica.^11^

Geralmente, as duplicações são alterações genéticas menos graves que as deleções no genoma, de forma que a ausência de CNVs é provavelmente mais patogênica. A eliminação de CNV pode ser mais prevalente em indivíduos com transtornos genômicos e, assim, confere um maior potencial de risco para a patogenicidade da síndrome, quando observada como uma alteração *de novo* em um indivíduo afetado.^12^

No presente estudo, foi realizado o teste de aCGH no paciente 8, sendo detectada uma microalteração cromossômica no braço longo do cromossomo 6, a deleção de segmento intersticial de 2,8 Mb, em 6q25.2-q25.3. O segmento afetado no cromossomo 6 contém vários genes, e deleções que superpõem esse mesmo segmento genômico já foram descritas em outros pacientes com quadro clínico variável. A deleção detectada inclui, entre outros, o gene ARID1B (614556), associado à DI.^13^

Ao se observar CNV de significado clínico incerto, deve-se primeiramente avaliar se ela é herdada ou *de novo*. O teste dos genitores é de grande importância para a determinação da patogenicidade da maioria das CNV, podendo existir uma comparação do material do paciente e dos pais. Isso proporciona uma indicação se a CNV é herdada ou não. Sempre que possível, estudo com exames citogenéticos convencionais devem ser considerados, uma vez que podem fornecer informações sobre a distribuição cromossômica e o risco de recorrência.^14^

As CNV de significado clínico incerto não são observadas na população normal e possuem genes de função pouco conhecida ou RNA não codificante, e sua interpretação ainda é um desafio para os pesquisadores.^15^ Nesse contexto, o resultado do teste aCGH da paciente 1 mostrou a presença de uma CNV com a perda de um segmento de 337 Kb do braço longo do cromossomo 13, em 13q13.3. Não há descrições de alterações semelhantes neste segmento genômico em indivíduos da população em geral. Não é possível afirmar que a variante detectada seja responsável por seu quadro clínico, pertencendo à categoria de variantes designadas na literatura como de significado ainda incerto. O resultado do teste da paciente 1 excluiu como causa de seu quadro clínico as inúmeras síndromes de microdeleções ou microduplicações genômicas descritas na literatura.

Analisando os resultados dos pacientes 6 e 7, foram encontradas alterações menores do que as possíveis de serem detectadas ao microscópio óptico em exame de cariótipo convencional. No primeiro caso, foi detectada uma deleção no segmento intersticial de 49Kb do braço longo do cromossomo 22, em 22q12.3. No segundo caso, foi observada uma variante cromossômica de 450Kb, resultado de uma deleção intersticial no braço curto do cromossomo 12, em 12p11.23. Na literatura, ambas microalterações são consideradas com significado clínico incerto.

Ainda sobre a classificação das CNV, aquelas determinadas como benignas não possuem um significado clínico para o fenótipo do paciente e aparecem em aproximadamente 6% do genoma humano. Existem genes no genoma com uma ampla variedade em relação ao seu tamanho e ao número de repetições, de modo que pacientes com quadro clínico normal podem apresentar um maior número de cópias, dificultando a interpretação dos resultados.^16^ Um dos pacientes apresentou deleção intersticial de 644Kb em 10q21.1. Essa deleção não afetou genes conhecidos, sendo descrita como uma alteração benigna, não causando efeitos deletérios ao indivíduo.

A crescente quantidade de informações coletadas pelos diferentes bancos de dados deve permitir estabelecer relação entre determinada CNV e uma possível condição patogênica com precisão crescente.^15^

No presente estudo, foi encontrado um maior número de microdeleções comparado ao de microduplicações. É mais provável que deleções no genoma sejam causadoras de um fenótipo alterado do que as duplicações. Geralmente microduplicações causam um fenótipo mais brando no portador.^17^

Em casos de pacientes com atraso no desenvolvimento psicomotor ou deficiência mental, nos quais o exame de cariótipo foi normal, recomenda-se a utilização da técnica de aCGH para auxílio no diagnóstico. Por conta da complexidade dessa metodologia, a interpretação do resultado em alguns casos pode ser dificultada. Recomenda-se, então, que a interpretação do resultado seja realizada com o apoio de um profissional especialista em genética, por meio de aconselhamento genético.^18^

## CONCLUSÃO

A análise de aCGH permitiu uma taxa considerável de detecção de anomalias cromossômicas que não foram detectadas na análise convencional por bandeamento cromossômico ou no exame de cariótipo.

Neste estudo, a caracterização do perfil genético por aCGH nos pacientes com deficiência intelectual e dismorfias possibilitou complementar o diagnóstico etiológico.

Apesar da difícil interpretação, o aCGH mostrou-se um método sensível, podendo ser utilizado como metodologia complementar em diagnósticos de doenças genéticas. Esse diagnóstico é importante em pacientes com etiologias raras ou ainda desconhecidas.
